# Biographical Feature: Benjamin A. Pinsky, MD, PhD

**DOI:** 10.1128/jcm.00198-26

**Published:** 2026-05-14

**Authors:** Erik Munson

**Affiliations:** 1 Wisconsin Clinical Laboratory Network Laboratory Technical Advisory Group, Madison, Wisconsin, USA; Vanderbilt University Medical Center, Nashville, Tennessee, USA

## TEXT

**Figure:**
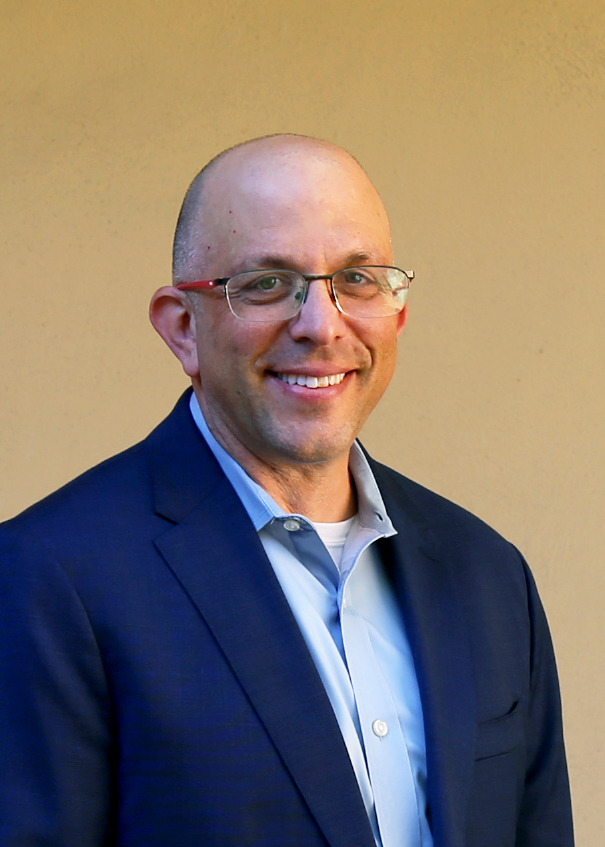


The following is a paraphrased excerpt from pleasantries exchanged at the beginning of a January video conference platform-aided conversation between a clinical virologist in California and a clinical microbiologist in Wisconsin (warning: highly relevant, groundbreaking scientific thoughts follow):

California clinical virologist: “I grew up in a suburb of Detroit. I guess that you’re familiar. You’re in the Midwest.”

Wisconsin clinical microbiologist: “(shivering) I am. It’s about 15 degrees right now, so I am very well familiar with that.”

California clinical virologist: “Yeah, yeah. I’m very happy to be in California. I should turn my background off, and you can see it’s nice and sunny and pretty warm out.”

The California clinical virologist was compassionate enough not to completely “rub in” his meteorologic good fortune on his discussant, and retained his virtual background of San Francisco’s Golden Gate Bridge throughout the conversation.

As illustrated in the previous text, attributes such as “funny,” “witty,” “very kind,” “self-effacing,” and “patient” are used (in part) by colleagues to describe the character of Benjamin Pinsky, MD, PhD, the recipient of the 2025 American Society for Microbiology (ASM) Award for Research or Leadership in Clinical Microbiology. Now affiliated with the Stanford University School of Medicine, Department of Pathology, and Division of Infectious Diseases and Geographic Medicine, Dr. Pinsky was born and raised in Michigan. His father was a practicing attorney, and his mother was an educator who dedicated a number of years to being a stay-at-home mother to Dr. Pinsky and his sister.

Dr. Pinsky reflected on his much younger days, professing a consistent interest in science, specifically biology and medicine. In a way, he downplayed his selection of an undergraduate institution, “So I thought I was going to go to the University of Michigan with most of my peers. I did go on college tours with my dad back in the day, and we went to see some of the “Ivys” and some of the small liberal arts colleges on the East Coast.” With respect to the consideration of these non-Midwest schools, Dr. Pinsky deadpanned, "Well, if I’m going to do this, I might as well try to get into this one (see next paragraph), so that seemed cool. Obviously, when you visit there as a kid from the Midwest, it’s very impressive. I mean, most of the “Ivys” are, but this one in particular. I guess that I was somewhat seduced by that and thought, ‘Well, let’s give it a go and see if I can convince them that I am worthy of attending.’”

The aforementioned “flyer” application was to Harvard University. Pinsky graduated from the institution in 1996 with *magna cum laude* honors and recalled, "Going into medicine was my intention, so I took the pre-Med courses. Biochemistry was my concentration and as part of a concentration, you do a thesis. As a result, I became interested in basic research.” At the time, Craig Crews, PhD (now faculty at Yale University) completed doctoral studies at Harvard under the tutelage of the late Dr. Raymond Erikson (another Midwesterner) and served as Dr. Pinsky’s concentration advisor. The connection between Pinsky and Dr. Erikson was forged and further cemented by interest taken by Pinsky in an undergraduate course on signal transduction taught by Dr. Erikson. Dr. Erikson was a co-recipient of the 1982 Albert Lasker Basic Medical Research Award (sometimes referred to as “America’s Nobels”) for being the first scientist to isolate the protein product of an oncogene from cancer-causing retroviruses and developing a model to facilitate investigation of additional oncogenes. The *src* oncogene of avian sarcoma virus, through the kinase activity of its protein product p60src, was determined to diminish cell capacity to respond to regulatory signals (1, 2). Dr. Pinsky related, “I worked in [Dr. Erikson’s] laboratory for my undergraduate thesis and actually stayed in his lab after graduation for a couple of years (3), which was great. It was a really great experience working with all of the scientists there. I really enjoyed all of that lab work, and I couldn’t decide whether I wanted to do a PhD or an MD. I actually didn’t decide, I just did both. At that point, you have all of the time in the world.”

Dr. Pinsky completed graduate work at the University of Washington in Seattle, Washington; a PhD in 2005 in the Program in Molecular and Cellular Biology and an MD in the Medical Scientist Training Program. In his recollection of that career path, Dr. Pinsky naturally began by deadpanning. “I figured I’d been on the East Coast for long enough, so I applied to schools in the Midwest and on the West Coast. I loved Seattle—I think that I went there on the one sunny day that they had that year.” The tone of the conversation turned a bit more serious as Dr. Pinsky stated that his decision was largely based on expanding his basic research knowledge base and skill set. “My PhD was with Sue Biggins, and that was basic cell biology on budding yeast. I worked on another kind of kinase (4), so it continued the signal transduction type of work that I was interested in. Of course, I used microbiology to study it, but at that point, I wasn’t really thinking about infectious diseases.” Sue Biggins, PhD, now Professor and Director, Division of Basic Science at the Fred Hutchinson Cancer Center, recalled her first interaction with Dr. Pinsky, “He asked if he could rotate in my lab. He had done much more reading about the lab than the average rotation student, and he had clearly thought a lot about what he wanted to get out of graduate school. He actually wanted to switch from a very well-known lab to my lab, which was just starting. This made me a little worried about his judgment, but then, I never questioned him again once I saw what good decisions he made in the lab.” To that end, other attributes afforded to Dr. Pinsky by colleagues include “brilliant,” “pragmatic,” “determined,” and “exceptional foresight.”

Dr. Pinsky moved south along the West Coast to the Stanford University School of Medicine to begin residency training in pathology in 2007. He remarked, “When I started residency, I thought that I was going to be a molecular pathologist. That was one of the reasons why I went to Stanford; they had a great fellowship in that (side note: Dr. Pinsky completed this fellowship in 2009–2010). I had worked with Ray Erikson and Sue Biggins on projects that were in a way related to human cell replication. Even the yeast work was on how chromosomes segregate and was somewhat related to cancer. So, I thought that I would be a molecular pathologist.” Ellen Jo Baron, PhD (5), Professor Emerita of the Stanford University School of Medicine Department of Pathology, recalled that Dr. Pinsky “was elected as Chief Resident, during which time he totally reorganized the Residency Training program to be more comprehensive and initiated several new and valuable components. So suddenly, he was on everyone’s radar as a strong leader.”

Dr. Pinsky chuckled over the fact that his first project during residency involved “being roped into something automated in the core laboratory.” However, a junior pathology faculty member at the time, Niaz Banaei, MD, invited Dr. Pinsky to collaborate on a laboratory-developed molecular diagnostic assay to identify members of the *Mycobacterium tuberculosis* complex (6). “I worked on this straightforward PCR assay, validated it, and then later that year, it’s up and running to characterize these clinical specimens.” This was the first of 27 collaborative published scholarly works for the two scientists. Added to this was the tutelage of the then-clinical microbiology laboratory director, Dr. Baron. Early on, these two scientists collaborated on developing a *Staphylococcus aureus mecA* assay for inpatient use (7) prior to the advent of commercial assays.

These residency experiences impacted Dr. Pinsky. “I got really interested in infectious disease when I started my residency training—totally hooked on microbiology and virology. I could use all of the molecular biology skills that I had gained during my PhD, and I could apply them directly to clinical diagnostic training. Working with Niaz and Ellen Jo was what really got me hooked on the profession of microbiology and how it could impact clinical decision making and patient care.”

Esther Babady, PhD, Clinical Microbiologist at Memorial Sloan Kettering Medical Center, in her nomination of Dr. Pinsky for the aforementioned ASM award, wrote that “Dr. Pinsky is an innovative clinical scientist. His laboratory is an international model of excellence in infectious diagnostics. He is a sought-after thought leader in clinical virology, being invited at the regional, national, and international level to discuss innovative approaches to diagnostics of infectious diseases with a focus on viral diseases.” Alexander McAdam, MD, PhD, Editor in Chief emeritus of the *Journal of Clinical Microbiology* and current ASM President, wrote that “emerging viruses are the common theme of his research and his work is marked by its timeliness and high quality. Some of his most important work was done during the COVID-19 pandemic, including work on the immune response to COVID-19 infection and immunization, viral pathogenesis, and sample pooling (8, 9) to address limited testing resources.” Steven Fuong, MD from the Stanford University School of Medicine Department of Pathology added that Dr. Pinsky “has been at the forefront of diagnostic testing for other emerging and re-emerging viral infections, including the Zika virus, monkeypox virus, influenza A H5, and measles.”

Dr. Pinsky’s transformation to clinical virologist occurred toward the end of his pathology residency and, in his opinion, was linked to events of 2009 influenza A virus H1N1 (10, 11). “It was exciting to be at the forefront of a newish pathogen at that time, and something that could cause a pandemic. I was able to take advantage of my expertise at bringing on these molecular tests; at the same time, that’s when most of clinical virology was transitioning to a much more molecular specialty.” Moreover, Dr. Baron wrote, “he published a paper (12) documenting the long-term carriage of influenza virus in the gastrointestinal tract of an infected patient. This paper was among the most influential in forming today’s accepted and ubiquitous practice of testing stool and sewage to assess community levels of several viruses, including influenza, polio, and coronaviruses.”

Beyond the conversion of clinical virology from a serologic- or culture-based field to a molecular specialty field (13, 14), Dr. Pinsky opined about major advancements in the clinical virology field, including those related to automation, virus quantitation, syndromic diagnostic panels, and introductory virus sequencing, then continued by describing potentially unmet needs. “I’m very interested in viruses that cause undifferentiated systemic febrile illness that are frequently found in returning travelers but can be endemic in the United States as well (I am particularly fond of dengue, chikungunya, and arboviruses). These are challenging in that the clinical presentation overlaps with numerous viruses and numerous pathogens (it doesn’t have to be a virus). It’s a very non-specific syndrome; it’s not localizing. Second, for many of the arboviruses and arthropod-borne viruses, the window for molecular diagnosis is very narrow, so that makes it challenging.” In many of these scenarios, serodiagnosis may either be prone to cross-reactivity or not available. Dr. Pinsky further noted that understanding more about the biology of the individual virus (i.e., the course of infection) could improve its respective diagnostic modalities.

Dr. Pinsky was asked to wear his prediction hat for the short- and long-term futures for clinical virology and quickly retorted, “I was asked to look into the crystal ball a lot during COVID, and I said the same answer. ‘I don’t know; my crystal ball is cloudy.’” He viewed the application of various sequencing technologies as a potential advancement in the field. “We’ll see how that turns out. Certainly, those have been available for a while yet have not gained widespread adoption.” Limitations to broad acceptance may include a lack of current understanding on how to use these genomic data in a clinically actionable fashion and obtaining results in a timely fashion. The conversation extended to applications of metagenomic testing (15) or microbiome analysis in terms of clinical course. Dr. Pinsky offered the prospect of “host response type assays (“omics”) and how one uses these in infectious diseases. Certainly, some have been commercialized, but how one is going to use them has not necessarily been well described or widely accepted.” He specifically cited metabolomics as a potential future means to diagnose or prognose infectious diseases. Dr. Pinsky concluded, “I’m not sure which one of those will be the one that pans out, but there’s certainly a lot of cool stuff out there. We’ve tried some metabolomics, so I’m learning how to use a mass spectrometer.” While Dr. Pinsky professed cordial respect for his chemistry colleagues, he does envision a future in which clinical microbiologists and clinical virologists do oversee the aforementioned testing advancements. Responsibilities could range from results interpretation to training artificial intelligence models to interpret test results.

The obligatory SARS-CoV-2 retrospective begins with one perspective from Dr. Baron. “When the COVID pandemic began, Dr. Pinsky moved into a local hotel near the Stanford Virology Laboratory during the week so that he would not waste valuable time commuting and could spend the bulk of his time leading the laboratory viral testing efforts.” As a result, “he developed among the first, if not the first, private laboratory molecular test system for SARS-CoV-2 that received emergency use authorization from the United States Food and Drug Administration, and then began testing samples from all over northern California to help inform the public health response to the pandemic, in addition to identifying which patients required isolation and/or treatment…[for some time], the testing laboratory was the primary source for northern California.” Keith Jerome, MD, PhD, Head of the Virology Division in the University of Washington Department of Laboratory Medicine and Pathology reminisced on his own experience formulating a laboratory-developed test (LDT) for SARS-CoV-2, “as soon as the LDT rules were relaxed, we were ready to go live with our testing—and our second question was, ‘who else is ready to go with an LDT?’” And the answer was Ben’s lab in Stanford! We were so proud at that point to claim him as one of our own, and to point to his prescience in preparing for COVID-19 despite the initial pushback from regulators.”

Dr. Pinsky’s impetus for expedient development of an LDT for SARS-CoV-2 was based on his past experiences with 2009 influenza A virus H1N1, which he characterized as a “pandemic virus that spread globally very rapidly,” and Zika virus. Collectively, Dr. Pinsky realized that “public health does a great job at epidemiology and monitoring. But when there are these big outbreaks, I feel like they need the help of the clinical laboratories to meet the demands. I think that was where we could help. We could provide additional capacity and also perhaps move more nimbly than the government could.” Dr. Pinsky additionally took note of the extraordinary rapid spread of SARS-CoV-2 in China, likely the byproduct of its status as a novel, respiratory infection. “I had conversations with one of our faculty, Steven Foung, who is a blood banker and a hepatitis C expert. He is involved in the Stanford Blood Center, and the Stanford Blood Center has always been very early to test (going back to human immunodeficiency virus) for new pathogens. So he and I both were very concerned about what was going on in China. He was very encouraging that we should have the assays up and available both for patient care but also to protect the blood supply—that turned out not really to be the case for SARS-CoV-2, but at the beginning, you don’t know.”

Dr. Pinsky went on to share additional personal insight, epiphanies, and potential lessons learned upon looking back to the early diagnostic days of the SARS-CoV-2 pandemic. “I think that the most important lesson was just the preparedness and being early to have diagnostics and just how much of an impact that can make on patients and on a healthcare system—in that, being able to keep a healthcare system functioning. It was a bit surprising how when there are new things, it seems like we frequently delay and folks are very reluctant to commit to developing diagnostic capacity in the case that it’s not used. It’s a balance. Is that a wasted resource or should we try? When do we make that decision? I think that’s really challenging.” Dr. Pinsky surmised that events of the SARS-CoV-2 infection pandemic have made clinical microbiologists, clinical virologists, and laboratory directors more attuned to this decision of “flipping the switch”; at the same time, he correctly ascertained that “there’s certainly COVID fatigue. ‘We don’t want to deal with another issue like this.’ So, I think in that sense, there’s some reluctance to really flip that switch.”

A second COVID-19 laboratory topic that certainly rings true to laboratory staff and purchasing agents is reagent and consumables procurement. “Before the pandemic, I never once thought about supply chain…not a single time…it became so important that first year of the pandemic, and now it’s part of the vernacular. It’s just very interesting when you’re in crisis mode, there are folks who really raise their level and just are amazing and others who really cannot. It is interesting how it brings out the best and worst in individuals and in groups.”

With an eye toward the future, Dr. Pinsky stressed the importance of coordinated efforts between clinical laboratories, hospital systems, public health authorities, and governmental agencies, with clear and transparent communication. “It started off really chaotic and eventually improved. That was really great, and hopefully, the next time that there is something like this, we’ve learned those lessons and those communication pathways will be much clearer.”

Dr. Pinsky has readily translated clinical accomplishments in the field (including more than 260 peer-reviewed publications, 20 book chapters, and 60 invited presentations) into leadership roles in the field. Steven Specter, PhD, Professor Emeritus, University of South Florida Morsani College of Medicine, wrote that Dr. Pinsky’s “leadership abilities help him achieve success both in the research environment and in organizational activities. His leadership in the Clinical Virology Symposium (Symposium Organizing Committee membership since 2014; Chair since 2023) and in the Pan American Society for Clinical Virology (President-elect or President since 2022) has helped advance virology by bringing informative and provocative programming that allows colleagues to advance the discipline.” Dr. Pinsky has further served as Co-Editor in Chief of the *Journal of Clinical Virology* since 2018; Co-Editor of the *Clinical Virology Manual*, Fifth Edition; and Section Editor for *Manual of Clinical Microbiology*, 13th edition. He has further contributed expertise to the College of American Pathologists House of Delegates (2018–present), Next-Generation Sequencing Project Team (2014–2017), and Microbiology Resource Committee (2012–2017). Governmental contributions have included membership on the United States National Academies Forum on Microbial Threats (2023–2025) and the Centers for Disease Control and Prevention Infectious Diseases Laboratory Working Group in 2019.

Such scholarly work, diagnostic and clinical acumen, and leadership role have translated into a number of recognitions. Dr. Pinsky was selected for the ASM Distinguished Lecturer program in 2020–2022. He was awarded the Diagnostic Virology Career Achievement Award by the Pan American Society for Clinical Virology in 2022. In 2025, Dr. Pinsky was bestowed with the Immunology and Infectious Disease Division Outstanding Achievement Award by the Association for Diagnostics & Laboratory Medicine and was elected as a Fellow of the American Academy of Microbiology. Dr. Babady provides this succinct summary of the contributions of Dr. Pinsky to clinical microbiology, particularly clinical virology: “Dr. Pinsky is a leader and expert in our field. There are only a handful of well-known and trusted clinical virologists in our field, and if you ask any clinical microbiologist to name them, Dr. Pinsky would be on EVERYONE’s list.”
